# A technology-enabled multi-disciplinary team-based care model for the management of Long COVID and other fatiguing illnesses within a federally qualified health center: protocol for a two-arm, single-blind, pragmatic, quality improvement professional cluster randomized controlled trial

**DOI:** 10.1186/s13063-023-07550-3

**Published:** 2023-08-12

**Authors:** Job G. Godino, Jane C. Samaniego, Sydney P. Sharp, Douglas Taren, Alexandra Zuber, Amy J. Armistad, Amanda M. Dezan, Azure J. Leyba, Janna L. Friedly, Aaron E. Bunnell, Eva Matthews, Maureen J. Miller, Elizabeth R. Unger, Jeanne Bertolli, Alison Hinckley, Jin-Mann S. Lin, John D. Scott, Bruce B. Struminger, Christian Ramers

**Affiliations:** 1 Laura Rodriguez Research Institute, Family Health Centers of San Diego, 1750 5Th Ave, San Diego, CA 92101 USA; 2 Center for Wireless and Population Health Systems, UC San Diego, 9500 Gilman Drive, Dept. 0811, La Jolla, CA 92093 USA; 3 Herbert Wertheim School of Public Health and Longevity Science, UC San Diego, 9500 Gilman Dr., La Jolla, CA 92093 USA; 4 Exercise and Physical Activity Resource Center, UC San Diego, 9500 Gilman Drive, Dept. 0811, La Jolla, CA 92093 USA; 5 Department of Pediatrics and Nutrition, University of Colorado, 13001 East 17Th Place, Aurora, CO 80045 USA; 6 Ata Health Strategies, LLC, 1537 D Street NE, Washington, DC, 20002 USA; 7 Project ECHO, University of New Mexico Health Sciences Center, 1650 University Blvd NE, Albuquerque, NM 87102 USA; 8 Department of Medicine, University of Washington, 1959 NE Pacific St, Seattle, WA 98195 USA; 9 Rehabilitation Clinic, Harborview Medical Center, 410 9Th Ave, Seattle, WA 98104 USA; 10 National Center for Emerging and Zoonotic Infectious Diseases, U.S. Centers for Disease Control and Prevention, 1600 Clifton Rd, Atlanta, GA 30333 USA; 11 School of Medicine, UC San Diego, 9500 Gilman Drive, Dept. 0606, La Jolla, CA 92093 USA; 12 School of Public Health, San Diego State University, 5500 Campanile Dr, San Diego, CA 92182 USA; 13 Global Hepatitis Program, Clinton Health Access Initiative, 383 Dorchester Ave, Boston, MA 02127 USA

**Keywords:** Post COVID-19 conditions (PCC), Long COVID, Myalgic encephalomyelitis (ME), Chronic fatigue syndrome (CFS), Post-infectious fatiguing illnesses (PIFI)

## Abstract

**Background:**

The clinical burden of Long COVID, myalgic encephalomyelitis/chronic fatigue syndrome (ME/CFS), and other post-infectious fatiguing illnesses (PIFI) is increasing. There is a critical need to advance understanding of the effectiveness and sustainability of innovative approaches to clinical care of patients having these conditions.

**Methods:**

We aim to assess the effectiveness of a Long COVID and Fatiguing Illness Recovery Program (LC&FIRP) in a two-arm, single-blind, pragmatic, quality improvement, professional cluster, randomized controlled trial in which 20 consenting clinicians across primary care clinics in a Federally Qualified Health Center system in San Diego, CA, will be randomized at a ratio of 1:1 to either participate in (1) weekly multi-disciplinary team-based case consultation and peer-to-peer sharing of emerging best practices (i.e., teleECHO (Extension for Community Healthcare Outcomes)) with monthly interactive webinars and quarterly short courses or (2) monthly interactive webinars and quarterly short courses alone (a control group); 856 patients will be assigned to participating clinicians (42 patients per clinician). Patient outcomes will be evaluated according to the study arm of their respective clinicians. Quantitative and qualitative outcomes will be measured at 3- and 6-months post-baseline for clinicians and every 3-months post assignment to a participating clinician for patients. The primary patient outcome is change in physical function measured using the Patient-Reported Outcomes Measurement Information System (PROMIS)-29. Analyses of differences in outcomes at both the patient and clinician levels will include a linear mixed model to compare change in outcomes from baseline to each post-baseline assessment between the randomized study arms. A concurrent prospective cohort study will compare the LC&FIRP patient population to the population enrolled in a university health system. Longitudinal data analysis approaches will allow us to examine differences in outcomes between cohorts.

**Discussion:**

We hypothesize that weekly teleECHO sessions with monthly interactive webinars and quarterly short courses will significantly improve clinician- and patient-level outcomes compared to the control group. This study will provide much needed evidence on the effectiveness of a technology-enabled multi-disciplinary team-based care model for the management of Long COVID, ME/CFS, and other PIFI within a federally qualified health center.

**Trial registration:**

ClinicalTrials.gov, NCT05167227. Registered on December 22, 2021.

**Supplementary Information:**

The online version contains supplementary material available at 10.1186/s13063-023-07550-3.

## Background

The clinical presentation, severity, and outcomes of coronavirus disease 2019 (COVID-19), which is caused by severe acute respiratory syndrome coronavirus 2 (SARS-CoV-2), are highly heterogeneous [1]. While some patients recover quickly, others suffer from persistent symptoms collectively known as Post-COVID Conditions (PCC) or Long COVID, which could be identified as early as 4 weeks after infection [2]. At present, scientific understanding of the diagnoses, phenotypes, and epidemiology of PCC is evolving [3, 4]. The number of patients experiencing PCC is increasing as acute COVID cases continue to rise as new variants occur and the public decreases prevention activities and behaviors. Many patients are presenting in primary clinics with a wide range of health consequences that are present four or more weeks after infection of SARS-CoV-2. In many patients, the reported persistent symptoms are highly debilitating, whether they arise secondary to detectable organ system damage or are of unclear etiopathogenesis [5]. Several of the most commonly reported persisting symptoms of PCC are also typically present in patients diagnosed with myalgic encephalomyelitis/chronic fatigue syndrome (ME/CFS) and other post-infectious fatiguing illnesses (PIFI) [6]. These include fatigue, post-exertional malaise, and cognitive impairment, among others. Disability resulting from PCC is now covered under the Americans with Disabilities Act [7]. Given clinical similarities in the constellation of symptoms between ME/CFS and PCC, the current clinical knowledge of ME/CFS has been beneficial in the approach to management and care of patients with PCC. Similarly, approaches to improving the clinical care of patients with PCC may also provide insights into the management ME/CFS and other PIFI. Taken together, there is a critical need to advance understanding of the effectiveness and sustainability of innovative approaches to the clinical care of PCC, ME/CFS, and other PIFI.

One potential approach is to utilize a technology-enabled multi-disciplinary team-based care model centered on case consultation and peer-to-peer sharing of emerging best practices (i.e., teleECHO (Extension for Community Healthcare Outcomes)) to support the management of complex cases associated with PCC, ME/CFS, and other PIFI. The teleECHO approach has previously been used in the management of Hepatitis C and HIV infections, and it has expanded into a wide variety of health care contexts^.^ [8, 9]. A typical teleECHO session includes a clinician presenting de-identified patient cases to specialists who then mentor the presenting clinician and others as they learn to manage patients with complex conditions [10]. These case-based discussions are often supplemented with short didactic presentations to improve content knowledge and share evidence-based practices [11]. An important goal of a teleECHO program is to provide primary care providers access to medical experts from a variety of domains who commit to long-term support and engagement in immediate synchronous interactions. Given that this approach is case-based, interactive, and occurs in real-time, it has a set of distinct advantages to the traditional practice of sequential in-person specialty referrals (e.g., cardiology, pulmonology, and neurology) to address complex cases. This may be particularly helpful for cases in which the etiopathogenesis of PCC, ME/CFS, and other PIFI is unclear and evolving, as multiple specialist perspectives may be generated and discussed with the treating primary care clinician. In contrast, the emerging archetype of brick-and-mortar PCC clinics includes a combination of referrals to specialties (e.g., infectious disease, neurology, cardiology, nephrology) that are typically out of reach in low-resource, community-based primary care settings where the majority of high-risk patients will be treated [12–14].

Although teleECHO represents a potentially promising approach to enhancing the complex care of patients, empirical evidence on the effectiveness of this approach to improve patient and clinician outcomes is limited. TeleECHO programs have rarely been evaluated for both effectiveness and implementation outcomes, and evaluations often lack (1) use of randomization, (2) inclusion of both quantitative and qualitative patient- and clinician-level outcomes, and (3) multiple, well-characterized comparison groups (i.e., counterfactuals) [15, 16]. Thus, there remains a need to generate high-quality evidence that a teleECHO program can effectively enhance the management of complex cases associated with PCC, ME/CFS, and other PIFI while maintaining a high-level of sustainability within a community-based primary care setting.

### Objective

The primary objective of the present research is to determine the effectiveness of FHCSD’s Long COVID and Fatiguing Illness Recovery Program (LC&FIRP) on clinician- and patient-level outcomes. LC&FIRP is comprised of a teleECHO program focused on multi-specialty case consultation and peer-to-peer sharing of emerging best practices to support management of complex cases associated with PCC, ME/CFS, and other PIFI. Our secondary objective is to determine the feasibility, acceptability, and sustainability of LC&FIRP. This study is intended to provide a fuller understanding of the potential impact of innovative technology-enabled multi-disciplinary team-based care models in low-resource, community-based primary care settings.

## Methods

The SPIRIT reporting guidelines were used in the development of this protocol [17]. The completed SPIRIT figure for this protocol can also be found in Table 1 for clinicians and patients. No committees have been created or engaged in this trial. Multiple principal investigators JG and CR are responsible for all aspects of the trial. They oversee staff engaged in recruitment, data collection, management, and analysis. Any required and unplanned protocol amendments will be reported to the Institutional Review Board (IRB) and updated in the trial registration.

**Table 1:** The overview of the study period including primary care provider (PCP) schedule of enrollment, interventions, and assessments

	**Study period**									
	**Enrollment**	**Allocation**	**Post-allocation**							**Close-out**
**Timepoint**	**0**	**0**	***3 M***	***6 M***	***9 M***	***12 M***	***18 M***	***24 M***	***30 M***	** ≤ *37 M***
**Clinician enrollment:**										
**Eligibility screen**	X									
***Informed consent***	X									
**Allocation**		X								
***Patient enrollment:***										
***Eligibility***	x									
**Broad Consent**	x									
**Allocation**		x								
**Interventions:**										
***[Intervention A]***			X	X	X	X	X	X	X	
***Patients A***			x	x	x	x	x	x		
***[Intervention B]***			X	X	X	X	X	X	X	
***Patients B***			x	x	x	x	x	x		
**Assessments:**										
***PCP baseline survey***	X									
***PCP follow-up survey***			X	X	X	X	X	X	X	X
***PCP interviews***										X
***Patient baseline survey***	x									
***Patient follow-up survey***			x	x	x	x				

### Partners

This study is funded by the Centers for Disease Control and Prevention (CDC) and is being conducted by a consortium of practice-based and academic institutions that include FHCSD, Ata Health Strategies, Project ECHO University of New Mexico Health Sciences Center (UNM), University of Colorado Denver School of Medicine (UoC), and University of Washington (UW).

### Study design

We will evaluate LC&FIRP using an effectiveness implementation hybrid type 2 design [16]. Specifically, we will conduct a two-arm, single-blind, pragmatic, quality improvement, professional cluster, randomized controlled trial in which 20 consenting clinicians across primary care clinics at FHCSD will be randomized at a ratio of 1:1 to either participate in (1) weekly teleECHO sessions with monthly interactive webinars and quarterly short courses or (2) monthly interactive webinars and quarterly short courses alone (a control group). All clinicians will have the option to receive Continuing Medical Education (CME) credit for the weekly teleECHO sessions and monthly interactive webinar. More details on the content of the weekly teleECHO sessions, monthly interactive webinars, and quarterly short courses are provided below. Clinicians will participate in LC&FIRP up to 37 months and throughout participation, 856 FHCSD patients diagnosed with PCC, ME/CFS, or other PIFI will be assigned to the participating clinicians (approximately 42 patients per clinician). Patient outcomes will be evaluated according to the study arm of their respective clinicians. Quantitative and qualitative outcomes will be measured at 3-, 6-, 9-,12-, 18-, 24-, and 30-months post-baseline for clinicians and for patients at 3-, 6-, 9-, and 12-months post assignment to a participating clinician. We will concurrently conduct a prospective cohort study to compare the two patient populations resulting from the randomized controlled trial with a third patient population resulting from enrollment at University of Washington’s Post-COVID Rehabilitation and Recovery Clinic (https://www.uwmedicine.org/specialties/post-covid-rehabilitation). An overview of the study period for clinicians and patients can be found in Table 1.

### Setting

Funded by the US Department of Health and Human Services’ Health Resources and Services Administration, Federally Qualified Health Centers (FQHCs) across the USA are responsible for providing health care services to low-income populations [18]. Over 30 million Americans (1 in 3 people in poverty and 1 in 7 people who belong to a racial or ethnic minority group) receive medical care at an FQHC [18]. FHCSD is one of the ten largest FQHC health systems in the nation and provides medical care to more than 165,000 patients through more than 1 million visits annually. The vast majority of FHCSD patients are low-income and are members of a minority population: 87% live below 200% of the federal poverty level, 16% are homeless, 41% are uninsured, and 83% are racial or ethnic minorities, with Black and Latino patients comprising 9% and 59% of the patient population, respectively (both overrepresented relative to San Diego Country demographics).

FHCSD operates 58 sites throughout San Diego, including 22 primary care clinics that are located in federally designated Health Professional Shortage Areas, 18 behavioral health facilities, 2 mobile counseling centers, 8 dental clinics, 3 vision clinics, 2 outpatient and 13 integrated substances use disorder clinics, 3 medication assisted treatment sites, 3 homeless service centers, and a safety net pharmacy. When the first cases of COVID-19 were diagnosed in San Diego County, FHCSD quickly expanded in-house high complexity laboratory capabilities and deployed reverse transcription polymerase chain reaction (RT-PCR) testing for SARS-CoV-2 RNA with Food and Drug Administration emergency use authorization.

FHCSD has also fully deployed a nationally certified Electronic Health Record (EHR) systemwide, which allows for patient portal access and robust clinical outcomes tracking [19, 20]. It is linked with the FHCSD health system’s self-designed, web-based Clinical Management Information System (CMIS), which facilitates patient care processes, quality assurance, and evaluation functions. Because FHCSD’s systems were developed on a service-oriented architecture, they are highly adaptive to meeting FHCSD’s data collection requirements for clinic management and practice analysis and are uniquely able to add new services in a timely fashion. All of the FHCSD health system’s information technology infrastructure is located in a secure data center supported by a diverse broadband network (100–500 mbps) mostly comprised of Cisco network elements. The data center infrastructure is provided with virtual servers and/or high availability services to ensure availability of the systems on a five nines basis. All applications and data are scheduled for backup nightly (full and incremental) and supported by a robust business continuity plan. The EHR system at FHCSD includes SNOMED CT (Systematized Nomenclature of Medicine—Clinical Terms), as the basis of diagnosing. This is then cross-walked to International Classification of Diseases Tenth Revision (ICD-10) and used for administrative coding and for patients’ problem list. The problem list contains all problems (active, non-active) which are managed by the FHCSD health system’s providers. Importantly, an ICD-10 code for PCC has been implemented and FHCSD medical leadership has directed all providers to use the code to identify COVID-19 patients with symptoms lasting for more than 28 days.

Among low-income populations to date, there has been a concerning imbalance between the high incidence, morbidity, and mortality from COVID-19 (and other diseases) and the limited access to healthcare innovations and research. This has highlighted a long-standing and often disregarded problem characterized by underrepresentation of low-income and minority populations in biomedical research and a lack of readily available infrastructure to truly strengthen inclusion in research [21]. If this is not addressed through meaningful effort, then the safety, effectiveness, and sustainability of interventions and treatments derived from research that includes predominately privileged populations will likely have diminished benefits for low-income populations. Rigorous and just scientific research requires significant participation from all without undue burden or exclusion. The present research seeks to address this issue.

#### Participants

The target for weekly teleECHO sessions, monthly interactive webinars, and quarterly short courses is licensed health care professionals. Therefore, the present research will include professional clusters that will consist of primary care physicians, physician assistants, and nurse practitioners at FHCSD caring for patients who have had persistent symptoms and a decline in health-related quality of life associated with PCC, ME/CFS, and/or other PIFI. A total of 20 clinicians (10 in each study arm) will be enrolled and throughout participation, 856 FHCSD patients diagnosed with PCC, ME/CFS, or other PIFI will be assigned to those participating clinicians (42 patients per clinician). In order to achieve a similarly sized sample of patients among cohorts in the proposed longitudinal cohort study, we will include 428 patients from the UW Medicine’s Post-COVID Telehealth Clinic who have been diagnosed with PCC, ME/CFS, and/or other PIFI.

#### Inclusion criteria

Inclusion criteria for clinicians includes (1) being employed by FHCSD for clinical care delivery, (2) being a licensed primary care physician, physician assistant, or nurse practitioner, (3) being willing and able to care for patients who have had persistent symptoms and a decline in health-related quality of life associated with PCC, ME/CFS, and/or other PIFI, and (4) being willing and able to actively participate in LC&FIRP for up to 37 months.

Patients will be referred to LC&FIRP and assigned to a participating clinician if they (1) are 18 years or older, (2) have had persistent symptoms and a decline in health-related quality of life associated with PCC, ME/CFS, and/or other PIFI, and (3) have completed FHCSD’s Broad Consent and authorized their Protected Health Information to be used for research purposes. FHCSD Primary Care Providers use CDC guidelines to diagnosis patients with PCC, ME/CFS, and or other PIFI before referring them to LC&FIRP [22, 23].

#### Exclusion criteria

There are no exclusion criteria.

### Recruitment, enrollment, and study duration

FHCSD medical leadership will send an email invitation for optional participation in LC&FIRP to the approximately 200 eligible clinicians at FHCSD. Those who are interested and meet inclusion criteria will provide written informed consent, complete a baseline survey, and will be randomized to one of the two study arms. Study enrollment commences upon randomization. The duration of the study for clinicians is up to 37 months. Patients assigned to participating clinicians are expected to be engaged in LC&FIRP as directed by their clinician over the course of 12 months.

FHCSD patients who have had persistent symptoms and a decline in health-related quality of life associated with PCC, ME/CFS, and/or other PIFI may be referred to LC&FIRP by a participating clinician or by a non-participating clinician. Patients who take part in LC&FIRP will fall into one of the following four categories: (1) existing and new ICD-10 diagnosis of PCC and a laboratory confirmed SARS-CoV-2 infection, (2) existing and new ICD-10 diagnosis of PCC and no laboratory confirmed SARS-CoV-2 infection, (3) existing and new ICD-10 diagnosis of ME/CFS, and (4) other PIFI. Only those patients who have completed FHCSD’s Broad Consent and authorized their Protected Health Information to be used for research purposes will be allowed to take part in LC&FIRP. Once FHCSD patients are enrolled into the program they will be followed by participating clinicians for a minimum of 1 year. Patients who advise that they have moved out of San Diego County but remain in the state of California may choose to continue seeing their LC&FIRP primary care provider via telehealth appointments and participate in surveys. Patients who have moved out of the state of California may no longer see their assigned LC&FIRP primary care provider due to telehealth laws, but they may choose to continue participating in surveys.

### Randomization, allocation concealment, and blinding

After eligibility and consent are confirmed and a baseline survey is completed, participating clinicians will be randomized at a ratio of 1:1 to one of the two study arms. An electronic randomization list will be generated using the latest version of the statistical software platform R (version 3.3.2, http://www.r-project.org). The list will be securely integrated into the cloud-based Research Electronic Data Capture (REDCap) tool. Allocation will be concealed from all investigators and staff until the study group is assigned. Only the study manager and research assistants involved in the delivery of the intervention components will subsequently be made aware of allocation. It is not possible to mask participating clinicians; however, their patients will have no knowledge of their potential participation in weekly teleECHO sessions, monthly interactive webinars, and quarterly short courses. All staff who are involved in the collection of data and investigators who conduct analyses will remain blinded to allocation throughout the study.

### Retention and withdrawal

In order to enhance retention in the study, all participating clinicians will be offered CME credit at no cost for their engagement in weekly teleECHO sessions and monthly interactive webinars. Clinicians will also be offered the flexibility to temporarily pause participation in sessions as an alternative to fully withdrawing. Participation in this study is voluntary. Participating clinicians may decide not to participate or may leave the study at any time. This decision will not result in any penalty or loss of benefits to which they are entitled. Information that has already been collected may still be used, but no new information will be collected. The withdrawal reason and the withdrawal date will be documented.

Clinicians who go on a leave of absence (LOA), while participating, will not be contacted about the study. Patients assigned to participating clinicians on LOA will remain a part of that clinician’s LC&FIRP patient assignment. However, if the patient seeks follow-up care, they will be scheduled to see another clinician participant in the same intervention arm. Additionally, clinicians who terminate their employment with FHCSD, retire, or move departments and are unable to see primary care patients will be withdrawn from the study. All of the data that they contributed up to the point of withdrawal will be included in the research. Patients assigned to clinicians who withdraw from the study will be reassigned to another participating clinician in the same intervention arm. Clinician participants who choose to temporarily pause participation will not be assigned new patients but will continue to see existing LC&FIRP patients within their panel.

### Intervention(s)

**Fig. 1:**
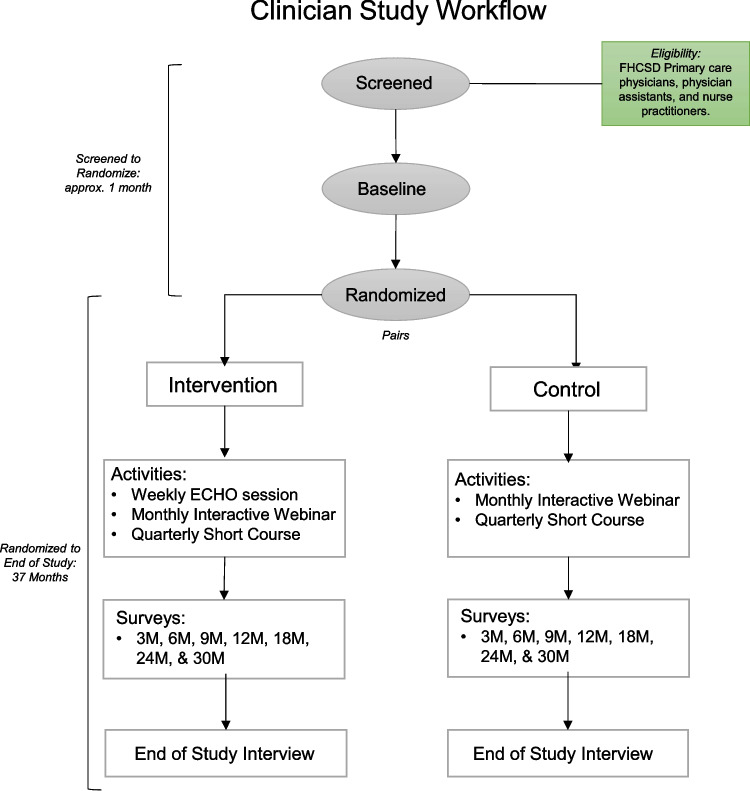
Flow of clinicians through the study

Of the 20 participating clinicians, 10 will be randomized to weekly teleECHO sessions. It is both impractical and inconsistent with high ethical standards to prevent the remaining 10 clinicians from accessing the dissemination of best practices via monthly interactive webinars and quarterly short courses, as these will be made available to providers across the USA. The subsections below provide more details on the intervention and an overview of the flow of clinicians through the study can be found in Fig. 1.

#### Weekly teleECHO sessions

The weekly teleECHO sessions will include a 10- to 20-min subject matter expert (SME)-led didactic presentation of emerging evidence-based best practices, followed by interactive review and discussion of 1–2 cases presented by clinicians for consultation with the multidisciplinary specialty panel. All weekly teleECHO sessions will offer CME credit for participants. De-identified case forms will be submitted by clinicians, and these will include patient medical and family history, current diagnoses, medications, and the clinician’s proposed inquiry to panelists. The composition of the specialty panel will include, but will not be limited to, national experts in pulmonology, neurology, neuropsychology, psychiatry, infectious disease, cardiology, rehabilitation medicine, and lived experience experts. Additional specialists will rotate and participate on a regular basis, and cases will be scheduled for presentation in an attempt to align with guest panelists. Upon conclusion of weekly teleECHO sessions, multidisciplinary recommendations will be distributed back to the presenting clinicians.

#### Monthly interactive webinars

We will convene monthly interactive webinars to rapidly disseminate findings and emerging best practices to a large-scale, national audience. The series will offer brief didactic presentations by SMEs, examples of models of care, and a facilitated question and answer session. The target audience will include clinicians participating in LC&FIRP, as well as primary care clinicians across the USA. The program will expand attendance by offering CME, Continuing Nursing Education (CNE), and Continuing Pharmacy Education (CPE) credit and engaging with organizations and associations outside the LC&FIRP partners through various dissemination strategies. These strategies include invitation to the monthly interactive webinar through existing listservs that reach these organizations’ and associations’ current members, as well as collaborative efforts in cross promotion on the LC&FIRP specific Project ECHO web page. Project ECHO will send an announcement for each webinar via email using a mass mailing platform and include the following information: title of Project ECHO program, session date and time, registration link, agenda, topic, speaker presentation slides (if available), and CME accreditation statements. In addition, Project ECHO will maintain the LC&FIRP specific Project ECHO web page and post agendas, video recordings, and resources shared during the monthly interactive webinars.

#### Quarterly short courses

Key findings from weekly teleECHO sessions will be consolidated into singular topics and delivered in quarterly short courses. These quarterly short courses will be formatted using multiple learning modules with the use of presentation slides and videos online that are accessible asynchronously. Following each module, there will be a set of questions learners will answer to review the material. There will be a post-course quiz at the end of each course with a score of 70% resulting in the printing of a certificate of completion. Each course will take less than an hour to complete. The target audience will include clinicians participating in LC&FIRP and primary care providers across the USA. Similar to the monthly interactive webinar, these quarterly short courses will be disseminated through existing partner channels, as well as organizations and associations collaborating with all project partners. To partake in the quarterly short course, learners must register through the University of Colorado’s (UoC) web page. Once published online, the quarterly short course will remain available until the next quarterly short course is published.

## Measures

### Clinicians

#### Quarterly survey

Data will be collected from all clinicians participating in the LC&FIRP, both intervention and control groups. Data collection is designed to document reported changes to provider knowledge, self-efficacy, and clinical behavior as a result of participating in the intervention or control conditions. The objectives of documenting these changes are to (1) help explain effects of the intervention on patient outcomes, including by comparing providers in the control arm based on reported changes to knowledge, self-efficacy, and behavior and (2) answer the co-primary implementation research question of this effectiveness-implementation trial: was the intervention implemented effectively and was it acceptable and feasible to implement among providers outside of the study context?

Data will be collected using electronic, web-based surveys, administered through REDCap managed by FHCSD. Surveys will be administered quarterly, to align with the collection of patient outcome data during the first 12 months. This frequency will also facilitate providers’ recall of specific changes to practice or behavior or improvements in knowledge in the last reporting period. Afterward, clinician survey data will be collected at the 18-, 24-, and 30-month mark. We anticipate the survey to take 8–12 min for each provider and comprise approximately 30 questions. The survey will remain open for a select period and reminder emails will be sent to encourage compliance.

Survey constructs and question formats are drawn from a deep body of existing research evaluating the impact of ECHO models on clinicians’ outputs and outcomes [15]. Survey methods and constructs were also studied among all randomized controlled trials of clinician-level outcomes globally captured in the Health Care Provider Performance Review, a multi-year systematic literature review process of global health worker performance evaluations [24]. To measure knowledge and self-efficacy, providers will be asked to state their relative agreement with statements related to their care of PCC patients, measuring from “strongly agree” to “strongly disagree” [15]. This 5-point response format will be held constant across survey knowledge and self-efficacy statements, to allow for an index to be created for use in more exploratory analyses. Open-ended response fields will be provided to some questions to allow providers to document specific examples of practice changes. Lastly, constructs probing overall provider satisfaction and utility with content will be measured to inform the acceptability and feasibility of the intervention.

#### Weekly teleECHO sessions

During the last 10 min of each weekly teleECHO session, intervention clinicians will be asked to follow a link to a web-based survey, loaded in REDCap. This semi-structured survey has three objectives: (1) to gather data required for the participants to receive CME credit, (2) to gather data useful for assessing feasibility and utility of the teleECHO model, and (3) to gather data on format and content of the sessions to guide program improvement. CME credit is offered by Project ECHO, University of New Mexico (UNM) to intervention providers who attend each session. To be eligible for this credit, clinicians are required to complete 20 post-session questions that are universal for every teleECHO session, which explore session relevance, achievement of course objectives, pace and delivery of the session content, and what participants liked most or least about the session. Additionally, we ask clinicians to rate their knowledge of the session content before and after the session, to explore knowledge gains, and if and how they intend to apply course content to their care of patients with PCC, ME/CFS, and other PIFI patients.

Making time within the session for this survey and making these questions mandatory for CME credit is expected to result in better survey compliance. The survey will last approximately 10 min and we will leave the survey available for a short time after the session to help clinicians complete the survey who might have been interrupted for any reason. If survey compliance is under 50%, a reminder email will be sent in a timely manner prior to the survey closing. Intervention clinicians’ survey responses will not be strictly confidential, as the questions are required to be documented by individuals for CME credit. With every survey, we will encourage clinicians to be candid in their responses, so that we can improve the ability of the teleECHO session curriculum to meet their needs. While this lack of anonymity presents a risk for bias, we will have the opportunity to assess similar constructs of utility and satisfaction in the quarterly surveys of participating clinicians, which will be strictly confidential and de-identified. This will help us to assess any bias and take further measures to reduce bias if needed.

#### Monthly interactive webinar

Data collected regarding the monthly interactive webinar will include webinar attendance and post webinar surveys for all clinicians participating in the Randomized Control Trial. Project ECHO UNM will offer CME, CNE, and CPE credits to all LC&FIRP participants and external learners for each monthly interactive webinar. This post-webinar survey will include a standard set of 23 questions that the Project ECHO UNM team requires for attendees to receive educational credits. The objective of collecting monthly interactive webinar data is to (1) understand the relative participation by study clinicians in monthly interactive webinars as a means to interpret differences in primary clinicians’ outcomes and (2) to support the evaluation of the feasibility, acceptability, and sustainability of the intervention. Survey responses will not be strictly confidential, as the questions are required to be documented by individuals for CME, CNE, or CPE credit. The survey will remain available after the monthly interactive webinar for 3 business days. If a participant misses that window and wants to claim credit, the ECHO survey team can reopen the survey if it is within 30 days of the webinar. After clinicians complete the survey, they will receive a certificate via email.

#### Quarterly short course

Data will be collected passively on numbers of clinicians completing each quarterly short course modules. The active collection of data will include responses by the clinicians to knowledge-based questions after each module, and at the completion of each course, about the value of the sessions and courses. The post-quarterly short course survey will include 10 questions about the knowledge gained, attitude changes that resulted from participation, and potential plans for behavioral changes resulting from what participants have learned. Measures collected from the post-quarterly short course survey for LC&FIRP participants and other learners will mirror the monthly interactive webinar survey. The objective of collecting quarterly short course data is to (1) understand the relative participation by study clinicians in monthly interactive webinars as a means to interpret differences in primary clinicians’ outcomes and (2) to support the evaluation of the feasibility, acceptability, and sustainability of the intervention. In addition to collecting data immediately before and following participation in the quarterly short course, LC&FIRP participants in the study will be asked to provide their responses to surveys that are specific to the trial outcomes.

#### Patients

Upon assignment to a participating clinician participant, patients will be asked to share current demographic characteristics, symptoms, medical history, health history, and, if applicable, their initial acute COVID-19 experience. Patients will be asked to complete a variety of widely used and validated patient reported outcome surveys that will take an average of 30 min to complete and will be repeatedly collected at 3-, 6-, 9-, and 12-months. In addition, symptom history and, if applicable, initial acute COVID-19 experience questions will be repeated during quarterly surveys to measure any changes over the 12-month follow-up period. Given the diverse nature of symptomatology, the patient cohort will first be characterized using descriptive statistics on domains of dysfunction (e.g., neurologic, cardiac, pulmonary, mental health). Surveys will include (1) Patient-Reported Outcomes Measurement Information System (PROMIS)-29, (2) Health Assessment Questionnaire (HAQ), (3) PROMIS Dyspnea Functional Limitations Short Forms, (4) PROMIS Applied Cognition Abilities and General Concerns Short Forms, (5) Patient Health Questionnaire (PHQ-9), (6)Generalized Anxiety Disorder (GAD)-7, (7) self-reported medication use, and (8) CDC ME/CFS Symptom Inventory Grid Screener [25–28]. All patient data collected will be integrated into the clinical workflows to maximize the likelihood of consistent completion. To track and collect patient surveys, a registry will be created within CMIS which includes specific survey forms for each survey frequency. All surveys completed will be stored within EHR and accessible for participating clinicians to review. Surveys will be conducted via phone calls from FHCSD staff supporting LC&FIRP, with calls ranging from 30 to 90 min.

**Fig. 2:**
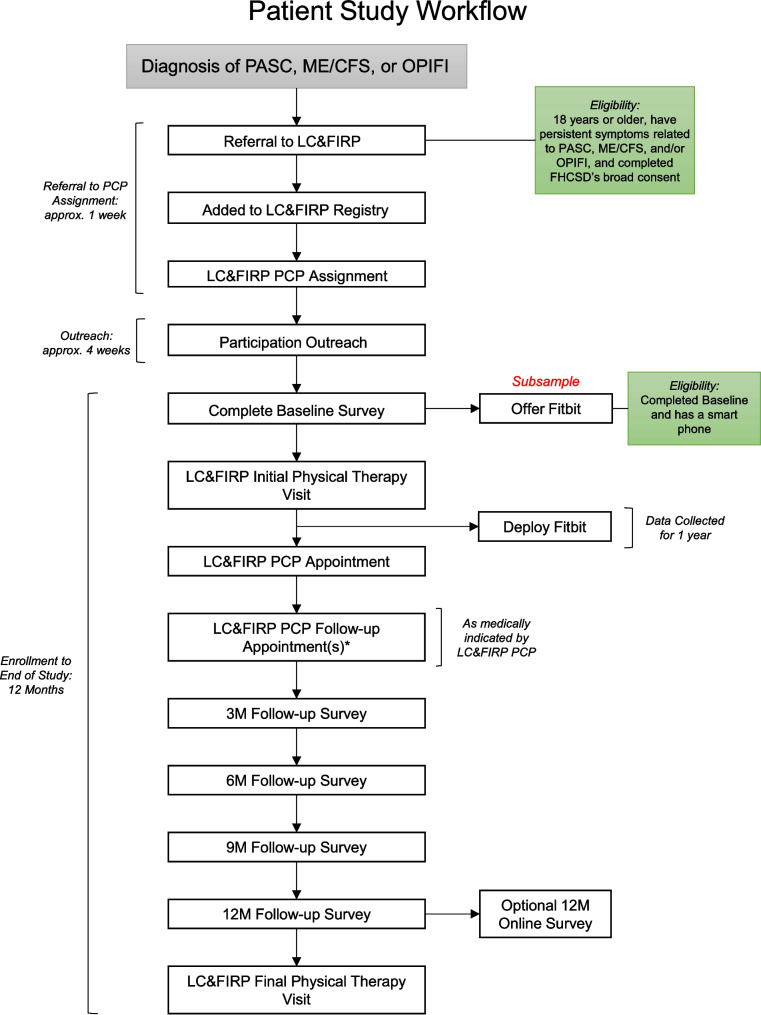
Patient flow through the study. *LC&FIRP PCP follow-up appointments may occur more than once

Each patient will be called a maximum of four times over the course of 4 weeks (once per week) to ensure the best possibility of achieving responses. Upon request, patients may request a copy of their survey responses. Patients who are unreachable for their 12-month survey or are unable to do the survey over the phone will be offered a shortened online version that includes the PROMIS-29 survey to be sent via text or email. An overview of the patient flow throughout the study can be seen in Fig. 2.

#### Physical therapy assessment

Upon completion of their initial assessment, patients will be asked to complete a physical therapy (PT) LC&FIRP visit. During their PT visit, patients will be asked to complete a variety of widely used and validated physical therapy assessments. These assessments include a 2-min step test, 30-s sit to stand test, grip strength, functional gait assessment, and balance tasks [29–33]. Due to appointment time constraints, not all assessments will be completed and additionally patients may decline or be unable to complete some assessments. All patient data collected during the PT visit will be stored in a specific form within the EHR and accessible to participating clinicians for review. Alternatively, patients may choose to decline participation in a PT visit, and this will not impact their ability to meet with their assigned clinician participant. Patients who completed an initial PT visit will be offered to schedule a final PT PCC visit during their 12-month follow-up survey call with FHCSD staff.

#### Wearables

A 20% (*n* = 172) subsample of the patients assigned to clinician participants will be offered a Fitbit device for remote monitoring. Patients will be offered a Fitbit randomly when agreeing to complete their PT visit or by clinician participants and PT providers who believe they may benefit from receiving one to help manage their symptoms. Patients will be asked to verify they have access to a smart phone and if they agree, they will be asked to wear their device as often as possible throughout the 12-month follow-up period. The Fitbit device will collect data on multiple physical activity metrics (e.g., intensity, steps, bouts of exercise), sleep metrics (e.g., time in/out of bed, sleep stages, sleep quality), physiology of the heart (e.g., beats per minute, heart rate variability), and, depending on circumstances, cardiorespiratory fitness. These data will be used to obtain a better understanding for the phenotype(s) of PCC, ME/CFS, and other PIFI. Data collected from Fitbit devices will automatically be uploaded to Fitabase, a Fitbit API partner who will provide a platform for FHCSD to view clean data ready for analysis. At the end of the patient’s 12-month review, their data will no longer be shared with Fitabase and they will be allowed to keep their device.

### Researchers

Researcher generated data will be collected by FHCSD staff supporting LC&FIRP through EHR chart review and survey data including clinical changes to practice, participation in interventions available, exposure to interventions, and patient experience. For all clinicians participating in the Randomized Control Trial, the changes to practice will be measured by the volume of total patient case load, volume of PCC and ME/CFS patient case load, and number of referrals made to specialists at the established intervals of 3-, 6-, 9-, 12-, 18-, and 30-months post baseline. For the 10 intervention clinician participants, assessment of changes to practice will include proportion of cases presented to a multidisciplinary specialist panel and number of specialist recommendations applied from case consultation. In addition, for the 10 intervention clinicians, we will measure the participation and exposure to topics in weekly teleECHO sessions, as well as participation in presenting case forms. For all 20 clinician participants, we will review participation in monthly interactive webinars and quarterly short courses and exposure to topics discussed in each, respectively. We will conduct an EHR chart review to measure the number of specialists seen by a patient and specialty types they were exposed to during their 12-month follow-up period. The objective of this data collection is to inform patient and clinician outcomes and support assessment of the feasibility, acceptability, and sustainability of the intervention.

### Semi-structured interviews

To inform our evaluation of the feasibility, acceptability, and sustainability of our proposed intervention, at the end of study, we will conduct 30-min interviews with the participating multidisciplinary specialists to obtain their perceptions of participation, satisfaction, and potential barriers to sustained participation. Sustainability will be influenced by how clinic administrators perceive participating clinicians’ performance and clinician’s perceptions of the expert consultation model. Thus, we will conduct 30-min qualitative interviews with both clinicians and clinic administrators to assess perceptions of change in clinicians’ productivity, patient panel composition, quality of care for complex cases, and influence on peers in the same clinic.

### Learners

Monitoring and evaluation of the monthly interactive webinar and quarterly short courses will include information from registered learners, webinar attendance data, and post webinar surveys. The demographic data will include but will not be limited to identifiable information about a learner’s affiliated organization, city of practice, clinical area of expertise, and patient populations served. The monthly interactive webinar and quarterly short course registration and attendance data will be collected passively through Zoom and REDCap Cloud, UNM, and UoC’s registration software, respectively. The attendance data will measure how many sessions each learner attended and the amount of time they attended. The objectives of this data collection are (1) to support the provision of CME and (2) to document the reach of the educational component of this intervention.

Project ECHO UNM will offer instant CME, CNE, and CPE credits to learners at each monthly interactive webinar. Learners will obtain the credits by filling out a brief post webinar survey accessed through a link in the email announcement and entered in the Zoom Chat toward the end of each webinar. Learners will fill out a survey with a standard set of questions required for accreditation as well as additional questions identified by collaborators as essential for the purposes of the program. Since the survey is required to receive educational credits, it must be personally identified and is therefore not anonymous. The survey will remain open after the monthly interactive webinar for a short time. After learners complete the survey, they will receive a certificate via email. Project ECHO will utilize a REDCap online data collection system to collect survey data for all post-webinar surveys. After each webinar, a formal feedback report will be provided to include a summary of responses and comments by session, aggregate CME feedback, total credits awarded, and attendees by type.

The quarterly short course survey will mirror the monthly interactive webinar survey. Any learner who is not a part of the randomized controlled trial will be asked to give consent for follow-up surveys. The quarterly short course survey responses, registration information, and attendance data for learners will be shared in an aggregate follow-up report with FHCSD.

### Data collection and generation methods

Clinicians will generate data via self-reported survey, clinical encounters, and engagement in teleECHO sessions. Data will be captured and stored using REDCap Cloud and iEcho. Patients will generate data via self-reported survey and results of clinical encounters. Data will be captured and stored within the EHR/CMIS at FHCSD. Researchers will generate data from review of clinical encounters with EHR and teleECHO sessions. Data will be captured and stored within EHR/CMIS at FHCSD, REDCap Cloud, and iECHO.

In addition, semi-structured interviews will be conducted with clinicians, multidisciplinary specialists taking part in teleECHO sessions, and clinic directors at FHSCD. All 30-min interviews will be recorded and transcribed, and transcriptions will be stored and processed using N-Vivo (QSR International software) for qualitative analysis. Learners will generate data via self-reported demographic information when registering for the monthly ECHO sessions and/or quarterly asynchronous short courses. In addition, learners will generate data via a self-reported survey upon completion of the monthly ECHO session and/or quarterly asynchronous short courses. Each of the pathways for the collection/generation of data will have a corresponding standard operating procedure (SOP) that trained staff will follow to ensure data quality.

#### Data privacy, management, and quality assurance

This study has been determined to be no more than minimal risk, given that risks are not greater than those encountered in the context of delivery and receipt of medical care. Any potential spontaneously reported harms will be dealt with in alignment with the policies and procedures of the covered entity, FHCSD. All data generated by clinicians and patients will be gathered using digital tools that allow for programming of data fidelity checks (e.g., preset ranges of acceptable values, error notifications for unexpected values, summary of skipped measures). Trained members of the research team will systematically review data generated by clinicians and patients in order to identify any issues that may be addressable in a timely fashion after data are meant to be collected. Data generated by trained research staff through chart review and administrative extraction will undergo manual fidelity checks by an additional member of the research team who will evaluate the data for completeness and accuracy.

The confidentiality of clinician and patient data is a top priority for the research team. Any information obtained about clinicians and patients during this study will be treated as strictly confidential to the full extent permitted by applicable law and in accordance with HIPPA regulations. Only trained members of the research team will have access to patient identifiers and data collected. All members of the research team will be trained to ensure confidentiality and adherence to standardized procedures. All research staff directly involved with the collection and storage of research materials will complete CITI training and internal FHCSD HIPAA and data security training. In addition, the research study will comply with policies established by FHCSD’s Data Security Plans. To ensure confidentiality, a research code number will be assigned to each participant and information that may identify them. The research code numbers will only be provided to qualified study investigators. Files linking names and other identifying information to data will be electronically saved using technology that prevents unauthorized individuals from accessing and understanding it. If a participant’s information is printed, it will be kept locked and accessible only to certified research staff. When study results are published no personally identifying information (according to HIPAA guidelines) will be revealed. All data will be collected and managed using secure, password protected, web-based tools that allow for programming of data fidelity checks (e.g., preset ranges of acceptable values, error notifications for unexpected values, summary of skipped measures). All entities involved in the present research (FHCSD, UNM, UoC, Ata Health, and UW) have access to such tools, like REDCap. REDCap provides an intuitive interface for data entry, audit trails for tracking data manipulation and export procedures, automated export procedures for seamless data downloads to common statistical packages, and procedures for importing data from external sources. Trained research staff will systematically review data generated by clinicians and patients in order to identify any issues that may be addressable in a timely fashion after data are meant to be collected. Data generated by trained research staff through chart review and administrative extraction will undergo manual fidelity checks by an additional member of the research team who will evaluate the data for completeness and accuracy. The present study is not exceptionally large or long term, and no planned interim analyses for efficacy or futility will be conducted. Therefore, a Data Safety and Monitoring Board will not be appointed.

### Statistical analysis

Analyses will be based on the intention-to-treat principle. All patients will be analyzed in the condition to which the clinician to which they were assigned was randomized regardless of compliance or fidelity. A “per-protocol” secondary analysis will be considered if compliance or fidelity of clinicians is a concern. All tests of significance will be two-sided and a *p*-value of 0.05 will be considered statistically significant. Group comparisons will be conducted on all the measured baseline patient characteristics to identify potential systematic differences between patients in each study arm. Identified patient characteristics that are not balanced will be used as covariates in the subsequent modeling of the data. Potential differential attrition between the two study arms will be compared and adjusted for in analyses. Analyses of differences in outcomes at both the patient- and clinician-level will include a linear mixed model to compare change in outcomes from baseline to each post-baseline assessment between the randomized study arms. Fixed effects will include the study condition, assessment, study condition-by-assessment interaction, baseline measure, and any unbalanced covariates. Assessment will be treated as a categorical variable, and an unstructured variance–covariance structure will be used. Results will be reported as point estimates (mean differences between groups) and interval estimates (95% confidence intervals). An intervention effect will be concluded if the *p*-value for the study arm-by-assessment interaction contrast in the model at the final assessment is statistically significant. This approach uses all available data and is robust to data missing at random (MAR).

Changes over time among clinicians in the intervention condition will be analyzed using generalized estimating equation (GEE) models with an exchangeable correlation structure. Depending on the variable distribution, we will either use log-binomial or Poisson models. The GEE approaches allow us to examine differences in subgroups by first including interaction terms into the models. This will enable us to explore if the intervention differentially impacts subgroups of learners and care teams defined by a variety of variables of interest (e.g., age, sex, location, etc.). Similar longitudinal data analysis approaches will allow us to examine differences in outcomes between cohorts at FHCSD and UW. In order to investigate the time to uptake of specialist recommendations between cohorts, a Cox proportional-hazards model will be applied. In addition, Kaplan–Meier curves and log-rank tests will be performed.

Data from the semi-structured qualitative interviews will be transcribed verbatim, transcripts will be uploaded into N-Vivo (QSR International software) for qualitative analysis. We will use a three-phased thematic analysis technique to code the responses using deductive and inductive codes. Authentication of key themes will occur by discussion and consensus with the research team.

### Sample size

To ensure that study has adequate power to determine the effectiveness of the intervention to improve outcomes of interest, we have powered our study on physical function after 12 months measured using the PROMIS-29. We believe that this will provide adequate power to examine differences in additional key outcomes (e.g., quality of life, anxiety, medication use, etc.) We calculated the sample size based on a two-sided, two-sample *t*-test at a significance level of 5%. The literature on minimally important differences in the PROMIS-29 domains suggests it is equal to 0.45–0.5 SD (4.5–5.0 points on a T-score metric). A 0.45–0.5 SD between group difference equates to a standardized effect size d between 0.45 and 0.5. As no similar cluster randomized controlled trial has been reported in the literature and Long COVID, ME/CFS, and other PIFI are heterogeneous, we have assumed that the intraclass correlation will be low (ICC = 0.1). Adjusting for this clustering of our primary outcome between 20 clinicians, we estimate that 600 patients (300 per condition, 30 per clinician cluster) will provide us with between 80.0% and 87% power. Assuming a loss to follow-up rate as high as 30% at the 12-month patient endpoint, we will aim to include approximately 856 patients (42 patients per clinician). In order to achieve balance in the proposed longitudinal cohort study, we will include 428 patients from the UW Medicine’s Post-COVID Telehealth Clinic.

## Discussion

This study will provide much needed high-quality evidence on the effectiveness of a technology-enabled multi-disciplinary team-based care model for the management of PCC, ME/CFS, and other PIFI within an FQHC setting, while simultaneously providing evidence regarding the feasibility, acceptability, and sustainability of the approach. Given that LC&FIRP includes a teleECHO program that is case-based, interactive, and occurs in real-time, it has a set of distinct advantages to the traditional practice of sequential in-person specialty referrals to address complex patient cases. This may be particularly helpful for cases in which the etiopathogenesis of PCC, ME/CFS, and other PIFI is unclear, as multiple specialist perspectives may be generated and discussed with the treating primary care clinician. It may also improve the targeted referral to local specialists to minimize unnecessary overuse of specialty care and reduce patient travel and time burden. We expect that the knowledge and confidence of participating clinicians will increase over time, that the cases presented to specialists will become more complex, and simpler routine cases will require consultation less frequently. This approach, which is clinician-focused, is nonetheless likely to result in more rapid provision of high-quality care for patients. We acknowledge that common clinical issues related to PCC, ME/CFS, or other PIFI will likely change over time. We consider our ability to measure this variation and explore its effect on a variety of clinician and patient outcomes as a strength. Taken together, findings from this research should inform how primary care providers and health system leaders support the clinical care of patients suffering from PCC, ME/CFS, and other PIFI.

## Trial status

This study was approved by Research Support Services at San Diego State University (Protocol Number: HS-2021–0241). This is protocol version 1.0 dated May 15, 2023. Recruitment began on November 30, 2021. The estimated study completion date is January 31, 2025.

### Supplementary Information

**Additional file 1.**

## Data Availability

The datasets analyzed during the current study and statistical code will be available from the corresponding author on reasonable request, as is the full protocol.

## Abbreviations

CDC: Centers for Disease Control and Prevention
CME: Continuing medical education
CMIS: Clinical Management Information System
CNE: Continuing Nursing Education
CPE: Continuing Pharmacy Education
EHR: Electronic Health Record
FHCSD: Family Health Centers of San Diego
FQHC: Federally Qualified Health Centers
GAD: Generalized anxiety disorder
GEE: Generalized estimating equation
HAQ: Health Assessment Questionnaire
ICD-10: International Classification of Diseases Tenth Revision
IRB: Institutional Review Board
LC&FIRP: Long COVID and Fatiguing Illness Recovery Program
LOA: Leave of absences
MAR: Missing at random
ME/CFS: Myalgic encephalomyelitis/chronic fatigue syndrome
PCP: Primary care provider
PHQ: Patient Health Questionnaire
PIFI: Post-infectious fatiguing illnesses
PROMIS: Patient-Reported Outcomes Measurement Information System
PT: Physical therapy
PCC: Post COVID-19 conditions
REDCap: Research Electronic Data Capture
RT-PCR: Reverse transcription polymerase chain reaction
SME: Subject matter expert
SOP: Standard operating procedure
SNOMED CT: Systematized Nomenclature of Medicine—Clinical Terms
teleECHO: Extension for Community Healthcare Outcomes
UoC: University of Colorado
UNM: University of New Mexico
UW: University of Washington
